# ‘A labyrinth with no way out’—the conceptualization of mental distress among Arabic-speaking refugee youth: a qualitative study

**DOI:** 10.3389/fpsyg.2026.1735835

**Published:** 2026-06-24

**Authors:** Youstina Demetry, Sarwar Kanabi, Jennifer Meurling, M. Ali Amiri, Gerhard Andersson, Shervin Shahnavaz

**Affiliations:** 1 Centre of Psychiatry Research, Karolinska Institute, Stockholm Healthcare Services, Stockholm, Sweden; 2 Habiliteringsmottagning Vuxna Lund, Region Skåne, Lund, Sweden; 3 Department of Psychology and Social Work, Mid Sweden University, Östersund, Sweden; 4 Department of Behavioral Sciences and Learning, Linköping University, Linköping, Sweden; 5 Department of Biomedical and Clinical Sciences, Linköping University, Linköping, Sweden; 6 Department of Health, Education and Technology, Luleå University of Technology, Luleå, Sweden; 7 HEI-Lab: Digital Human-Environment Interaction Labs, Universidade Lusófona, Lisbon, Portugal

**Keywords:** explanatory models, daily functionin, healthcare user perspective, mental distress, young refugees, ERG theory

## Abstract

**Background:**

Previous qualitative research suggests that the biopsychosocial explanatory models of mental illness are not always supported in non-Western cultures. This underscores the importance of exploring explanatory models of mental distress among hard-to-reach groups, such as refugees. The main objective of the current study was to explore what Arabic-speaking refugee youth and young adults conceptualize as their mental distress and if they experience lower functioning or if their mental distress relates to unmet needs in the daily life.

**Method:**

A qualitative study design was applied to analyze the responses to the Psychological Outcome Profiles Questionnaire (PSYCHLOPS). An inductive approach using content analysis was utilized to explore what Arabic-speaking refugee youth and young adults conceptualize as their mental health problem. Alderfer’s Existence-Relatedness-Growth theory was employed deductively to understand which needs are unmet in relation to their mental distress.

**Results:**

The identified problems fell into one of three main domains: challenges within the self, difficulties in exile and challenges in the encounter with ‘the other’. The mental health problems hindered existence needs and needs of relatedness, as described by the Alderfer’s existence-relatedness-growth (ERG) theory.

## Introduction

The term ‘hard to reach’ has been used to describe populations that are underrepresented in research (Shaghaghi et al., 2011). Refugees are considered one such subgroup, as they are not only underrepresented in clinical research but also underserved within mental healthcare services (Gubi et al., 2021; Wendler et al., 2005). In addition, refugees and other minority groups are frequently excluded from clinical studies (Redwood and Gill, 2013). Such exclusion has serious implications, including reduced generalizability, social injustice, health disparities and ultimately an increased long-term disease burden (Oakley et al., 2003).

The number of studies that have explored the perspectives of refugees on mental health problems is limited (Craig et al., 2009). Mental health problems are defined as difficulties that affect an individual’s cognitive, emotional and social abilities but not to the extent that criteria for formal diagnosis for mental disorders would be fulfilled (Fuller et al., 2000). In the context of migration and resettlement, mental health encompasses domains beyond the medical lens of psychopathology and include aspects of safety and security, social bonds and networks, justice, roles and identities, and finally existential meaning (Tomsfelt, 2021). This stands in contrast to the growing recognition of the importance of incorporating healthcare users’ perspectives as an essential component in the planning and delivery of healthcare services, as well as clinical research (Hansen et al., 2004). The limited knowledge among mental health practitioners regarding cultural concepts and explanatory models of mental distress further highlights the need to include refugees’ voices in both research and practice (Gupta and Bhugra, 2009; Hebebrand et al., 2016).

Previous studies suggest that the biopsychosocial explanatory models of mental distress do not fully capture experiences of mental distress across cultures (Aarethun et al., 2021; Hassan et al., 2015; Tanous et al., 2025). This is particularly evident in the initial years of post-migration, after which these models become complemented by alternative explanatory frameworks as time in exile increases (Mölsä et al., 2010). Explanatory models are strongly shaped by cultural factors, such as beliefs in demonic (*‘Jinn’*) possession, witchcraft and engagement with the occult (Leavey et al., 2016). Spiritual explanations, such as loss of religious identity and secularism also feature prominently as perceived causes of mental distress (Leavey et al., 2016). Another common culturally grounded explanation is the belief in the ‘evil eye’ (Alqasir and Ohtsuka, 2024). The concept rests on the idea that displaying wealth, well-being, or general satisfaction may attract jealousy and envy from others, which can subsequently lead to illness and misfortune (Alqasir and Ohtsuka, 2024).

Culture and context shape not only how psychological distress is experienced and interpreted, but also whether, when, and from whom individuals seek help (Alqasir and Ohtsuka, 2024; Deng and Rose, 2025; Borho et al., 2021). Help-seeking is, therefore, influenced both by cultural understandings of symptoms and by socially available responses to distress. For example, Syrian refugees in Germany have been reported to use healthcare services for somatic symptoms at higher rates than the general German population, suggesting patterns of help-seeking that may differ from those typically expected in the host context (Borho et al., 2021). In some cultural frameworks, symptoms that might be classified in Western cultures as depression or psychological distress may instead be understood as social, relational, or moral problems. As a result, individuals may turn to culturally meaningful coping strategies, such as seeking a romantic partner or consulting a religious leader, rather than formal mental health services. Similar issues arise in relation to trauma-related symptoms. When refugees have been asked about PTSD symptoms as defined by DSM-5, they have sometimes described these reactions as normal responses to war and displacement rather than as signs of mental disorder (Aarethun et al., 2021; Hassan et al., 2015). Given that war and forced displacement may become embedded in everyday life for those living in conflict settings, such responses may be understood as part of surviving extreme adversity rather than as inherently pathological (Gupta and Bhugra, 2009). This highlights the risk of interpreting distress exclusively through PTSD or trauma frameworks, thereby pathologizing responses that may be contextually understandable (Craig et al., 2009). Therefore, when meeting individuals who have experienced war, it is crucial to look beyond PTSD and trauma frameworks and consider what additional forms of distress they may be experiencing and what factors are most impacting their daily function.

Daily function is defined as the individual’s ability to carry out daily activities and perform their social roles without significant constraints (McCallum et al., 2019). Exposure to traumatic events or the presence of symptoms does not necessarily translate into impaired functioning. In fact, a study from Norway found an association between mental illness and functional impairment among Syrian male refugees, but not among Syrian female refugees (Nissen et al., 2022). Another study reported that female refugees did not display apparent signs of reduced psychological function, despite exposure to up to 18 traumatic events, including torture (Robertson et al., 2016). Instead, compromised functioning among female refugees was associated with post-migration stressors, such as perceived discrimination. Conversely, among male refugees, changes in family dynamics were associated with reduced functioning (Robertson et al., 2016). Despite these informative quantitative studies, a deeper understanding of the relationship between mental distress and functioning, from the perspective of individuals who have fled their home countries as children, remains scarce.

Considering the aforementioned findings, there is a need to explore young refugees’ own perspective on their mental health problems and whether these difficulties impact their level of functioning. Furthermore, if such an impact exists, in what ways do mental health problems relate to functioning. To explore functioning and unmet needs among Arabic-speaking young refugees, the Alderfer’s Existence-Relatedness-Growth theory (ERG; 23) will be applied (more details below).

Young refugees, belong to what is known as Generation 1.5 (Benesch, 2008). In one definition, Generation 1.5 is determined by age at arrival and refers to individuals who were born in their country of origin, migrated to the host country before the age of 12, and experienced their identity-formation years in exile (Benesch, 2008). Additionally, Bartley and Spoonley expand the arrival age range to include children arriving to the host country between the ages of 6–18 (Bartley and Spoonley, 2008). Other definitions suggest that belonging to Generation 1.5 is determined by whether an individual received formal primary education in the host country (Manchenko and Westerveen, 2019). Both age at migration and length of residence in the host country appear to influence the prevalence of mental health (Alegría et al., 2017; Breslau and Chang, 2006), with younger newcomers showing a higher risk of developing psychiatric illness (Breslau and Chang, 2006). Beyond the prevalence of mental illnesses, the “in-between” generation faces additional challenges, including negotiating autonomy within the family, navigating peer relationships that may require diverging from family cultural values, and forming an identity across at least two cultures (Turjanmaa et al., 2017). On the other hand, Generation 1.5 appears to differ from first generation immigrants in terms of acculturation and social outcomes (Rumbaut, 2004). Given the multifaceted challenges and opportunities facing 1.5 Generation, it is important to gain a deeper understanding of their experiences. To date, however, this subgroup has been largely overlooked in literature.

### Alderfer’s ERG theory

Alderfer’s ERG theory, developed between 1961 and 1978, was originally proposed to explain human motivation and has been widely applied in organizational contexts (Alderfer, 1989). The theory posits that individuals strive to satisfy three core needs: existence, relatedness, and growth (Alderfer, 1969). *Existence needs* include material and physiological requirements for survival and security. *Relatedness needs* involve desires that are fulfilled through relationships and social interaction. *Growth needs* refer to personal development and self-actualization; that is, “making creative and productive effects on oneself and the environment” (Robertson et al., 2016, p. 146), and are met by pursuing opportunities for advancement. Unlike Maslow’s hierarchy of needs, a key features of ERG is that these need categories are not hierarchal and may operate simultaneously; thus, needs may overlap across categories depending on how they are pursued (Wanous and Zwany, 1977; Maslow, 1943). This is particularly relevant to refugees, for whom existence needs co-occur with efforts to secure social integration, navigate new relationships, and maintain ties with their existing relationships. Thus, the pressure to satisfy existence, relatedness and growth needs is concurrent, and these needs are intertwined and are mutually dependent. In contrast to Self-Determination Theory, ERG theory explicitly incorporates material insecurity, central to pre- peri- och postmigration experiences, as part of the existence needs.

According to ERG, similar activities may fulfill different needs. For instance, seeking employment to secure financial stability reflects an existence need, whereas seeking a job to work with specific individuals reflects a relatedness need. Moreover, employment may simultaneously satisfy all three needs: existence (economic security), relatedness (social integration) and growth needs (personal development and contributing to society). ERG’s frustration-regression mechanism is related to the experiences of refugees, where structural factors during post-migration may redirect efforts away from growth needs toward intensified focus on existence needs (Alderfer, 1969; Wanous and Zwany, 1977). Similarly, satisfaction in one need domain can increase the salience of needs in another need (Alderfer, 1969; Wanous and Zwany, 1977).

The present study aims to explore the following research questions: (1) what Arabic-speaking youth and young adults in exile conceptualize as their mental health problems? and (2) If and in what ways do the conceptualized problems inhibit the youth’s functioning or ability to fulfill their needs?

## Materials and methods

### Study setting

The study was a part of a larger project aiming to examine the efficacy of a culturally adapted internet-based cognitive behavioral therapy targeting Arabic-speaking refugee youth and young adults with mild to moderate mental health problems (Demetry et al., 2026). Data were collected as part of the randomized controlled clinical trial, during an 8-month-period between end of November 2023-beginning of April 2024 and end of August 2024-end of December 2024. Upon providing informed consent, participants were directed to the online screening questionnaire. The randomized controlled clinical trial has received ethical approval through the Swedish Ethical Review Authority (dnr: 2022–04274-02).

### Participants

Participants were recruited through paid advertisements on Facebook and Instagram for a randomized controlled clinical trial. Participants provided their consent and registered to the study through the Raha Arabic Intervention page (Demetry et al., 2026), which is hosted on Linköping University’s Iterapi platform (Vlaescu et al., 2016). A total of 349 participants registered to the study and completed the screening questionnaire. Of these, 234 were excluded for: age, scoring below the cut-off score of the primary outcome measure (HSCL-25; 26) (Parloff et al., 1954), not completing a prescreening task (the trial module), or being unreachable for the assessment phone interview. For this analysis, individuals who do not have a refugee background were also excluded. The final analysis included responses from 115 participants. Table 1 presents the characteristics of the sample. The mean age of the participants was 25 years (range 17–29), and approximately 56% of the participants were males. Approximately, 84% of the participants originated from Syria, and 60% resided in Sweden. Nearly 60 % of our sample have been living in exile for 8–10 years, indicating that a large proportion had arrived as children. The sample is also organized in two cohorts: namely, Generation 1.5 and Generation 1. For the current study, we have defined Generation 1.5 as participants who have arrived in the host country between the ages of 6–18. Consequently, we define Generation 1 as participants who have arrived in the host country at the age of 19 or older.

**Table 1:** Demographic characteristics of the Arabic-speaking young refugee sample.

Demographic characteristics	*N* = 115 (%)
Age	
Mean (SD)	25.10 (2.97)
Min-max	17–29
Age at arrival	
Mean (SD)	18.42 (4.44)
Min-max	7-27
Generation 1.5 age at arrival	
Mean (SD)	15.10 (2.52)
Min-max	7-18
First Generation age at arrival	
Mean (SD)	22.32 (2.68)
Min-max	19-27
Gender	
Male	64 (55.7)
Female	50 (43.5)
Other	1 (0.9)
Country of birth	
Syria	96 (83.5)
Iraq	5 (4.3)
Egypt	2 (1.7)
Yemen	3 (2.6)
Palestine	6 (5.2)
Sudan	2 (1.7)
Stateless	1 (0.9)
Country of residence	
Sweden	69 (60)
Germany	36 (31.3)
Norway	3 (2.6)
Denmark	2 (1.7)
Sint Maarten	1 (0.9)
United Kingdom	1 (0.9)
Netherlands	1 (0.9)
Finland	1 (0.9)
Cayman Islands	1 (0.9)
Highest education level	
Secondary school	11 (9.6)
High school	38 (33.0)
University degree	61 (53.0)
Other	5 (4.3)
Employment status	
Student	74 (64.3)
Employed	22 (19.1)
Unemployed	13 (11.3)
Parental leave	1 (0.9)
Sick leave	2 (1.7)
Other	3 (2.6)
Years in country of residence	
0–3	34 (29.6)
4–7	16 (13.9)
8–10	59 (51.3)
More than 10 years	6 (5.2)
Migration status	
Asylum seeker	7 (6.1)
Temporary residency permit	40 (34.8)
Permanent residency permit	17 (14.8)
Citizenship	51 (44.3)

### Measurement

The Psychological Outcome Profiles (PSYCHLOPS) is a problem-based idiographic patient reported outcome measure (I-PROM), designed to assess psychological distress and changes in distress over the course of psychological interventions (Sales C. M. D et al., 2023). PSYCHLOPS exists in several versions corresponding to the different phases of psychological interventions. The present study used the pre-intervention version. The pre-intervention version consists of three domains and includes four items (Sales C. et al., 2023). The first domain focuses on the primary problem area(s) for which the patient/participant is seeking help and contains two free-text, person-generated items. The first item instructs participants to “Choose the problem that troubles you most”. The second item in this domain is similar to the first one except that it asks the participant to choose “another problem that troubles” them. The second domain concerns functioning and include one free-text item, person-generated item: “choose one thing that is hard to do because of your problem (or problems)”.

PSYCHLOPS was filled out in Arabic. For the analysis, the data were translated into Swedish by the first author. To assess semantic equivalence, a random subset of responses was back-translated into Arabic and compared with the original Arabic responses. Free-text data from both domains were used to address the present research questions.

### Data analysis

**Figure 1:**
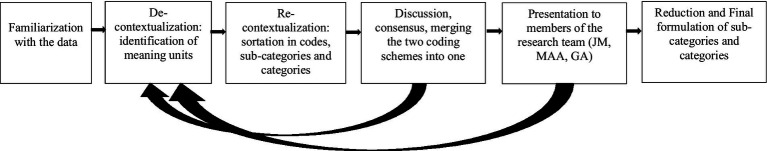
The analytical process.

To answer the first research question, an inductive approach utilizing a qualitative content analysis was implemented (Lindgren et al., 2020). Given the nature of the research question and the concise format PSYCHLOPS, where participants describe their problem areas and functioning in brief free-text responses, the analysis focused on the manifest content of the data (Graneheim et al., 2017; Kondracki et al., 2002). Manifest content refers to the “visible [and] obvious components” of the data (Vlaescu et al., 2016, p. 106). The analytic process consisted of three coding cycles, each involving both de-contextualization and re-contextualization of the data (Friberg et al., 2000) De-contextualization refers to separating concepts from individual respondents in order to highlight participants’ experiences related to the phenomenon of interest (Friberg et al., 2000). In practice, this involved condensing the data and identifying meaning units, which were coded independently by two of the authors (YD and SK). Re-contextualization refers to the process combining the meaning units into new patterns to gain deeper insights into the concept under study (Graneheim et al., 2017; Friberg et al., 2000). Line-by-line coding was conducted and meaning units were then sorted into codes, which were then grouped into subcategories and categories. The re-contextualization phase inferred a higher degree of abstraction compared to the de-contextualization phase (Lindgren et al., 2020; Graneheim and Lundman, 2004). The first cycle of analysis produced two separate coding schemes. After completing this cycle, the two coders compared their coding schemes and discussed discrepancies until reaching consensus. The two were then merged into a unified coding structure. A second cycle followed, in which the coders revisited both the meaning units, and the updated coding scheme. Members of the research team were subsequently invited to a presentation in which the coders shared the preliminary coding scheme and received feedback. Finally, the coders revisited the raw data, meaning units and coding scheme once more to refine and finalize the subcategories and categories (Rennstam and Wästerfors, 2018). An overview of the analytic process is presented in Figure 1.

To answer the second research question, a deductive approach was employed. The meaning units were sorted into subcategories, which were then organized into predetermined categories following Alderfer’s ERG theory (Alderfer, 1969). The findings are reported according to the standards for reporting qualitative research (SRQR; 39, see Appendix 1) (O’Brien et al., 2014).

### Researchers’ characteristics, approach, reflexivity

A pragmatist approach was adopted in the current study (Morgan, 2014). Following Morgan, pragmatism understands knowledge as grounded in experience, generated through inquiry into problematic situations, and evaluated in terms of the consequences it has for interpretation, action, and understanding (Morgan, 2014). The deductive strategy utilized to answer the second research question, was chosen not as a rigid test of theory, but as a structured and purposeful way to examine the data in relation to the research question. Thus, knowledge is understood as provisional and developed through engagement with participants’ accounts, researcher interpretation, and the research context. In this sense, reality is not understood as wholly subjective, but neither is it accessed independently of experience; instead, understandings of reality are continuously refined through inquiry and interpretation (Sol and Heng, 2022).

There are various ways of defining reflexivity. Olmos-Vega et al. (2023) definition was adopted here. They defined reflexivity as the practice of continuous self-critique and evaluating one’s subjectivity and the ways in which this may influence the research process. Participants were not known to any of the research team members when the data were collected. All authors have a background in clinical psychology and research experience in migration and mental health. The first and second authors were the primary analysts of the data. The third (JM) and fourth authors (MAA) knew the data well and were continuously involved in the analysis. These discussions contributed to new perspectives and refinement of categories. Conceptualization and categories were discussed in the research group before the first and second author settled on the final categorization.

After the initial screening, the first author (YD) was known to 54% of the participants due to conducting intake assessment interviews and later in her role as a therapist for participants who met the inclusion criteria. Co-authors SS, and MAA learnt to know about the participants through part-taking in the intake treatment conferences. The co-authors were, however, not known to the participants.

The first author (YD) is a 1.5-generation immigrant, born in Egypt and moved to Sweden at the age of eight. She has been surrounded by cultural idioms for mental health/distress, and she has observed patterns of culturally specific coping strategies. Relevant to this study, she worked as a clinical psychologist in Egypt between 2015 and 2018. While it was useful to have worked with Arabic-speaking patients, she made conscious efforts not to accept common assumptions regarding Arabic-speaking patients at face value. Furthermore, it was useful for her to have known the participants in her role as a therapist, as this at times contributed to a deeper understanding of their answers. With that said, efforts were made to distinguish when it was appropriate to allow the data analysis to be influenced by the author’s knowledge of the participants and when it was not. Finally, reflexivity was furtherly maintained through discussions with the research team members, self-awareness and self-critique.

The second author (SK) recently obtained his degree of Master of Science in Psychology in January of 2025 from Stockholm University. As SK is a second-generation immigrant with close experiences of immigration and Arabic-speaking culture, SK engaged in several structured reflexive procedures to actively acknowledge his own biases and assumptions regarding his knowledge of the participants throughout the data analysis, and, through these practices, enabled critical examination of his own preconceptions of the research topic. These reflexive procedures included participation in numerous research conferences, team briefings and individual meetings with the first author (YD); practices that encouraged critical feedback and furthering strengthening analytic transparency, for example while discussing one’s preconceptions of the research topic and thereby ensuring that the analysis conducted was grounded in the data rather than a personal stance. When appropriate, his lived experiences allowed him to approach the data analysis with deepened cultural awareness, which may have aided in a more contextually grounded reading of the data and its nuances.

## Results

### The inductive analysis of mental health problems

The inductive analysis of the problem areas described by the refugee youth and young adults resulted in the identification of 477 codes. The codes were abstracted into three categories (A-C) and ten subcategories (A1-A6, B1-B2, C1-C2) as shown in Table 2 (for a detailed coding scheme, see Appendix 2). The three categories identified were: (A) Challenges within the self (77%), (B) Difficulties in exile (17%), and (C) Challenges in the encounter with ´the other` (7%). Within the first category, six subcategories were identified: (A1) the inner apprehensions (29%), (A2) the diminished vitality (13%), (A3) the emotional turbulence (8%), (A4) the eroded self (11%), (A5) difficulties staying on track (27%), and (A6) the bodily strains (11%). Within the second category, two subcategories were identified: (B1) isolation in integration (71%), and (B2) socioeconomic demands (29%). Within the third category, two subcategories were identified: (C1) conflicts with others (53%), and (C2) the longing for connection and stability (46%).

**Table 2:** Categories, sub-categories, meaning units, and example quotes.

Category	Sub-categories	Example meaning units and/or codes	Example quotes
Challenges within the self	The inner apprehension	Worry, fear, rumination, traumatic experiences, anxiety, political stress, existential rumination	*“Anxiety, fear, panic attacks and depression occur all at the same time or during the same period, and the annoying thing is that sometimes I feel like nothing is worth worrying about and sometimes I feel very heavy and afraid of simple things.” – Male from Palestine, 19 years* *“Fear and worry” – Female from Syria, 22 years*
	The diminished vitality	Depression, loss of joy, indifference, hopelessness, meaninglessness	*“In general, I have lost the enjoyment of life, and I suddenly feel depression in the middle of a happy or supposedly happy or pleasant situations” – Male from Palestine, 28 years* *“Constant despair: it feels as if there is no hope in the future, as if I am a prisoner in my own mind, stuck and nothing can help me get out. Everything seems impossible to accomplish, and the worst part is that I know the problem lies within me, not in the world around me. I am constantly pessimistic and expect the worst consequences and possibilities.” – Male from Syria, 28 years.*
	The emotional turbulence	Anger, irritability, grief, feeling of emptiness, dissociation, jealousy	*“Jealousy. I am jealous of the romantic relationships I see around me, and I am jealous of my friends’ successful relationships. I am also jealous if my friends go out without me and when they do things together without me.” – Female from Syria, 25 years.* *“Anger attacks for no reason or over little things that I thought it must be hormonal problems because it seemed irrational, but it wasn’t because this has been going on for a long time and it is not linked to a specific time period.” – Female from Syria, 29 years.*
	The eroded self	Self-hate, low self-esteem, negative perception of self-actualization.	*“[my] feeling of disappointment, self-hate and negative thoughts” – Male from Iraq, 29 years*
	Difficulties staying on track	Concentration problems, lack of motivation, learning difficulties, procrastination, inner restlessness	*“Lack of concentration, forgetfulness and irregular sleep. This affects every detail of my life, the most important of which is my studies. It bothers me that I cannot pass my exams.” – Female from Syria, 21 years* *“I think I have ADHD and therefore suffer from several problems: forgetfulness, difficulty concentrating, difficulty with self-control and emotions, constant anxiety, easily distracted, which negatively affects my self-esteem.” – Male from Syria, 29 years*
	The bodily strains	Sleeping difficulties, lack of energy, tiredness, bad appetite	*“Extreme fatigue. I always feel tired and weak. As if I am drained. I do not have the energy to do anything for myself. I try to exercise and walk, but it rarely works. I feel totally depleted mentally and physically” – Male from Syria, 28 years*
Difficulties in exile	Isolation in integration	Involuntary loneliness, separation from loved ones, acculturation stress, subjective racism, language barriers,	*“Loneliness, lack of friends in exile” – Female from Syria, 26 years*
	Socioeconomic demands	Unemployment, economic difficulties, lacking daily routines, difficulties related to studies	*“My father always calls me to ask for money” – Male from Syria, 25 years* *“Career options” – Male from Syria, 21 years*
Challenges in the encounter with ´the other`	Conflict with ‘the other’	Family conflicts, negative perception of others	*“Anxiety, stress, fear, low self-esteem, difficulty changing my deeply rooted beliefs about what it feels like to be rejected. All of this was due to living with my family and being subjected to psychological pressure from them with their pathological relationship styles, which distorted my beliefs and drained me of energy and vitality. And because I still suffer threats from them today.” – Male from Syria, 19 years*
	The longing for connection and stability	Relationship conflicts, singlehood, imbalance in the romantic relationship,	*“I was used by someone whom I thought was my life partner” – Female from Syria, 29 years* *“When I broke up with my girlfriend, after having found her and experienced peace for the first time since I moved to [country of residence] and after I have finally found a home. But after the separation, I feel like I have lost my home, and I feel like I have become a stranger for the second time. This is what I think about almost 90% of the time” – Male from Syria, 26 years*
Unmet needs	Unmet existence needs	Studies, daily activities, work, sleeping, bad hygiene, bad appetite	*“Studies and work” – Stateless Male, 27 years*
	Unmet relatedness needs	Lack of communication skills, develop friendships, time for the family	*“I do not have the energy or time to meet my parents…” – Female from Syria 26 years*

#### Mental health problems organized by generation

To compare how mental health problems were conceptualized by Generation 1.5 refugee youth and young adults versus first-generation refugee youth and young adults, the category structure was compared across the two cohorts. As shown in Table 3, Generation 1.5 youth were more likely than first-generation youth to conceptualize their mental health problems in terms of inner apprehensions (A1; 34%), difficulties staying on track (A5; 33%), and conflicts with others (C1; 80%). In contrast, first-generation refugee youth were more likely to conceptualize their mental health problems in terms of the eroded self (A4; 18%), bodily strains (A6; 14%), and longing for connection and stability (C2; 73%). The two groups were similar across the other subcategories.

**Table 3:** The percentages of the identified categories and subcategories defined per cohort.

Categories and the associated subcategories	Cohort generation 1.5 (*n* = 62)	Cohort generation 1 (*n* = 53)	Whole sample (*n* = 115)
**Challenges within the self**	78	76	77
The inner apprehensions	34	24	29
The diminished vitality	11	15	13
The emotional turbulence	10	7	9
The eroded self	5	18	11
Difficulties staying on track	33	21	27
The bodily strains	8	14	11
**Difficulties in exile**	14	20	17
Isolation in integration	72	70	71
Socioeconomic demands	28	30	29
**Challenges in the encounter with ´the other`**	9	5	7
Conflicts with others	80	27	54
The longing for connection and stability	20	73	46

##### Challenges within the self

Challenges within the self were described as mental health problems that participants attributed to their inner being and personal abilities. The category is characterized by participants expressing a sense of responsibility for self-managing these problems. One participant wrote *“…I try to do everything despite how I feel”.* The quote reflects an endurance-oriented coping strategy, in which the participant prioritizes functioning despite ongoing mental distress. While this high sense of responsibility may preserve daily functioning in the short term, it can contribute to sustained distress in the long term. Maintaining short-term daily functioning may also limit the realization that professional help is needed. Other participants expressed how these internal challenges affected them across different situational contexts.

**Figure 2:**
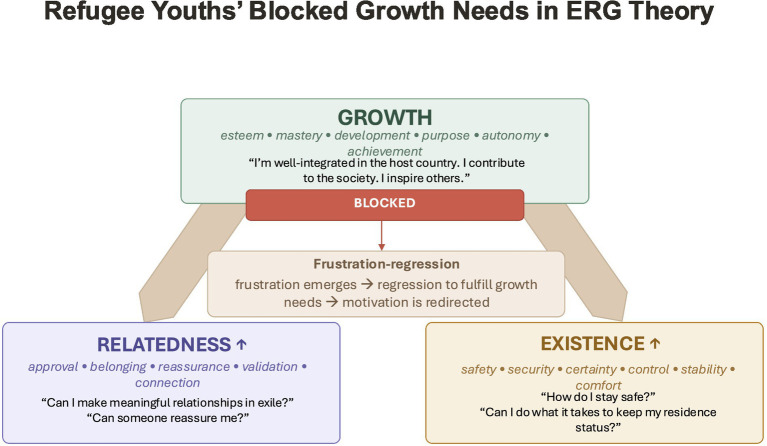
The categories and associated subcategories regarding the mental health problems expressed by the participants.

###### The inner apprehension

The most frequent responses within this subcategory included fear, rumination, anxiety and worry. One participant described anxiety and fear as *“a labyrinth with no way out”* (in Arabic: *Mataha min doun makhrag)*. Another participant described fear and worry as follows:

*I constantly worry. Sometimes I worry so much that I get panic attacks, with physical symptoms (tiredness, shakiness, sweatiness, and pale face) and sometimes I get dark depressive thoughts. The main reason is the fear of not getting a residency permit in [name of country of asylum] due to the Dublin regulation. Triggering factor: I constantly follow the news (almost every hour). – Male from Syria, 25 years, arrival age 24.*

The quote above illustrates worry, conceptualized as an inner apprehension, as a persistent form of mental distress. The participant explicitly attributes this internal experience to external stressors, namely uncertainty regarding residency status and continuous exposure to news. These external stressors are described not only as cognitive experiences, but also as somatic manifestations, including tiredness and shakiness. The quote further highlights how post-migration stressors, particularly structural and legal conditions, can impose a substantial psychological burden. Although the participant primarily frames his mental health problems in terms of worry, he also expresses depressive thoughts, indicating a broader impact of these stressors on his psychological health. Finally, the participant identifies frequent news monitoring as a trigger. Checking the news almost every hour, a potentially maladaptive coping strategy, temporarily alleviates uncertainty while simultaneously maintains the inner apprehension.

Other frequently mentioned problem areas included political stress, stress, existential rumination, and past traumatic experiences. For example, one participant described how the inability to forget past events affected their capacity to progress in life.

*[what troubles me is] my inability to let go of the past, my inability to progress and develop in the society and the fear I feel of most things – Male from Syria, 29 years, arrival age 24.*

The participant expresses a sense of entrapment in the past. Past events have disrupted the participant’s life trajectory, and he now experiences a tension between the desire to grow and the inability to progress. Another participant expresses rumination in the following manner:

*I never let myself rest mentally. I keep blaming myself for things, and I overanalyze everything I do. I don’t feel like a normal human being. – Male from Syria, 20 years, arrival age 11.*

The participant describes a sense of being stuck in a loop of self-blame and self-criticism. Apprehension is constant and is expressed as a state of inner tension, with no room for mental recovery. Ultimately, this loop has left the participant feeling a sense of alienation and disconnection from what it means to be a normal human being.

###### The diminished vitality

The most frequently mentioned problems in this category were depression and despair, as expressed by several participants. Participants who expressed their mental health problem as depression, usually did not elaborate further. The quote below illustrates how despair was expressed:

*I have lost the enjoyment of life, and I slip into a state of depression even in the midst of a happy situation; or what is supposed to be a happy or enjoyable situation – Male from Palestine, 28 years, arrival age 18.*

The participant expresses an inability to connect with enjoyable moments, suggesting that his capacity to experience enjoyment and pleasure is greatly diminished. The quote also indicates an awareness of the contrast between how situations are socially understood as pleasurable and how they are actually experienced by the participant. Lack of enthusiasm is expressed by another participant.

*A fading enthusiasm for life as a whole. – Male from Syria, 25 years, arrival age 16.*

The participant’s fading enthusiasm for life appears to shape his overall mode of experiencing the world, influencing how he perceives not only his present circumstances but life as a whole.

*In reality, I suffer from despair and the loss of ability to carry out a normal routine. I also think a lot and I have difficulties sleeping more than two hours. I suffer from psychological loneliness, not a social one. I think a lot. I look for jobs and at the same time I’m afraid of not being able to keep one. I procrastinate most of the things and then I regret it. I don’t know exactly what my problem is, but pessimism has become a dominating character in my life. I stay up late and sleep a lot from sunrise to midday. – Male from Syria, 21 years, arrival age 20.*

The above quote highlights a multi-layered distress that interferes with daily functioning. Explicitly, the participant expresses despair, an inability to maintain a daily routine, insomnia, loneliness and rumination. He also describes a procrastination cycle in which avoidance of tasks is followed by regret. Overtime, the disrupted routine and ongoing procrastination appear to have contributed to a pervasive sense of pessimism, leading to feeling stuck in time and unable to move forward.

###### The emotional turbulence

This subcategory included responses describing difficulties in emotion regulation. The most frequently mentioned emotion in this subcategory was anger, as illustrated by the quote below:

*I get anger attack over no particular reason or over small reasons. I thought it was hormonal problems because they seem irrational, but it is not because it has been going on for a long time and I can’t link it to a specific time in my menstrual cycle – Female from Syria, 29 years, arrival age 27*.

In the quote above, the participant initially attempts to make sense of these experiences through a biological and gender-specific explanatory model, attributing them to hormonal fluctuations. This reflects an effort to normalize or rationalize the emotional difficulty by linking it to bodily processes. Anger was also expressed by participants belonging to Generation 1.5:

*I get very angry over very little things – Female from Syria, 22 years, arrival age 14.*

Lack of emotions was also expressed by a couple of participants, as illustrated by the following quote:

*The feeling of emptiness...I sense that it is important to keep a distance from other people – Male from Syria, 20 years, arrival age 14.*

###### The eroded self

Living in a limbo with no clear idea of what the future holds and an inability to meet one’s personal goals, is described by many participants as contributing to an eroded sense of self. The most frequently mentioned code in this subcategory was low self-confidence, as illustrated by the following example:

*I have very low self-confidence even though I perform well. It makes me feel bad and I often question myself and my worth. The strange thing is that this happened after fleeing [from my home country]. I came to [name of the country of residence] when I was 18 and that was 10 years ago. Before that I was a very confident person. – Female from Syria, 29 years, arrival age 18.*

###### Difficulties staying on track

This subcategory comprises responses related to higher functions, such as lack of motivation, restlessness, and procrastination. A large proportion of participants described concentration difficulties as their main problem, as in the example below:

*In the past four years I have experienced big changes in my personality, my feelings, my way of thinking and my whole life…I can’t concentrate on anything, not even the phone calls with my parents. When I start a small task like warming up food for lunch, I leave the task before finishing it and I forget about it. I’m very distracted and my concentration is very weak. I was never like this before. I was ambitious, confident, achieved everything I wanted, and I had an energy that was admired by my surrounding…Now I don’t have energy even if I don’t do anything…” – Female from Syria, 21 years, arrival age 14.*

###### The bodily strains

Participants reported bodily symptoms, such as tiredness, loss of appetite, sleeping problems, whereof the most frequently mentioned symptom was loss of energy. One participant elaborated on this by writing:

*I don’t have enough energy to leave the house. I chose to study online so that I don’t have to leave the house and I haven’t visited my mom in more than four months even though she lives in the same town – Female from Syria, 26 years, arrival age 17.*

##### Difficulties in exile

Difficulties in exile were related to challenges faced post-migration. During pre- and peri-migration, reaching a safe place is often highly anticipated and idealized. The reality of post-migration hardships can therefore come as a surprise, shattering the previously held hopes of prospering and integrating. (1) isolation in integration, and (2) socioeconomic demands.

###### Isolation in integration

The most mentioned problem in this subcategory was involuntary loneliness. One participant wrote:

*Loneliness and lack of friends in exile – Female from Syria, 26 years, arrival age 17.*

Another shared difficulty in making friends in the host country.

*I don’t have friends, and I just stay at home. I have a hard time getting to know new people and I overthink. – Male from Syria, 29 years, arrival age 22.*

Here, the participant appears to ruminate over their ability to make new friends in exile. This reflects the notion that refugees are often required to rebuild their social networks entirely upon arrival in the host country.

###### Socioeconomic demands

Difficulties related to studies were the most frequently mentioned problems in this subcategory. In the context of migration, obtaining a degree is considered a doorway to a better future, and therefore holds a unique significance for the participants. This is illustrated by the following quote:

*The academic pressure of having to learn a new language under pressure and in a short period of time is difficult. This is to prepare for the [name of occupation] equivalency exam as well as the temporary residence permit. I’m forced to achieve all of this in just two years – Female from Syria, 26 years, arrival 25.*

The quote above reflects the pressure to achieve academically under significant time constraints, as the youth feel responsible for compensating for an involuntarily disrupted life trajectory. In this case, academic pressure may also be tied to the participant’s residence status. Consequently, academic achievement carries meanings beyond personal development or prosperity.

##### Challenges in the encounter with ‘the other’

Challenges in the encounter with the ‘the other’ was identified as another problem area and included two subcategories (see Figure 2). This category reflected situations in which ‘the other’ and/or interactions with ‘the other’ were described as contributing to the participant’s distress. The term ‘the other’ captures participants’ sense of alienation, even in relation to family members and close ones. Within this category, two subcategories were identified: (1) conflicts with the other and (2) the longing for connection and stability.

###### Conflict with others

One problem area in this subcategory was characterized by negative perceptions of ‘the other’, or negative perceptions of how ‘the other’ view them, as depicted in the following example:

*I have lost my passion, and I have a feeling that others don’t appreciate my existence – Female from Palestine, 26 years, arrival age 9.*

‘The other’ include even family members. A number of participants reported conflicts with family members as their main problem area, as illustrated in the following:

*Conflicts with the family; especially my mom. They have control over me, and they are dissatisfied with everything I do – Female from Syria, 17 years*, *arrival age 10.*

The quote illustrates how family relationships can become a source of psychological distress in the post-migration context. The participant describes persistent maternal disapproval and experiences her mother’s behavior as controlling. Such ongoing criticism may undermine the family’s role as a source of emotional support and safety. At 17, she is in a developmental period typically characterized by intensified identity exploration and increasing striving for autonomy and independence—tensions that can be challenging even in stable circumstances. For a young refugee girl, these developmental demands may be further compounded by post-migration pressures and the need to renegotiate family boundaries.

##### The longing for connection and stability

This subcategory captures a desire for emotional anchoring. Participants expressed a longing for connection and a sense of “being held” by something static. A romantic relationship is imagined as a stabilizing structure; that is, something that softens loneliness, and makes life feel more coherent and manageable. Conflict within these relationships can have a domino effect on other relationships and burden the soul. Bachelorhood was one of the problems described in this subcategory. One participant expressed the following:

*Approaching the age of 30 without marriage, children or stability. I feel that starting a family will not happen soon. I also worry about losing my mom and dad before I get to see them. – Male from Syria, 29 years, arrival age 26.*

The quote captures the participant’s sense of being “behind” as he approaches 30, with marriage and family formation presented as salient markers of adulthood. He evaluates his progress through culturally shaped expectations, linking personal success to achieving stability. From this excerpt alone, it is not possible to determine to what extent he attributes this disrupted life course to migration. However, his fear of losing his parents before he is able to see them appears directly connected to forced separation, and this time-pressured worry likely intensifies his psychological distress. Bachelorhood was a concern even among Generation 1.5:

*Loneliness and that I haven’t found a life partner yet – Female from Syria, 25 years, arrival age 16.*

The participant associates her loneliness with not having found a life partner. This reflects how emotional struggles may be interpreted through external circumstances, with the absence of a partner becoming a meaningful explanation for her distress. Another problem area in this subcategory was conflicts with the romantic partner. One participant described the conflicts with the romantic problems in the follow manner:

*The romantic problems with my ex-fiancé and problems with my sister-in-law have led to a bad relationship with my brother who lives here in [country of residence] – Female from Syria, 26 years, arrival age 25.*

In many Arab cultures, it is a common norm that a relationship is not formed solely between two individuals; rather, it is understood as a union between two families. Consequently, it is not sufficient to meet only the needs of one’s partner. An individual is also expected to engage with and accommodate the expectations of their partner’s entire family.

### The deductive analysis of unmet needs

To understand the impact of the identified problem areas on the needs and daily functioning of the young refugees, we analyzed the functionality domain in PSYCHLOPS using Alderfer’s ERG theory (Alderfer, 1969). The deductive analysis resulted in the identification of 98 codes. The codes were organized into one category and two sub-categories. The category was unmet needs. The two subcategories in this category were: (1) unmet existential needs and (2) unmet relatedness needs.

#### Unmet needs

##### Unmet existence needs

Unmet existence needs are characterized as material and physiological needs or desires that are important to the individual. Their importance is independent of the achievements of others in the person’s surroundings. For example, success in studying reflects an internal need rather than a desire to stand out from the crowd. The most frequently mentioned unmet needs within this subcategory were difficulties in managing studies, daily chores, and work. Challenges related to sleep, hygiene, and appetite were also described as unmet needs. One participant summarized her main challenge in the following way:

*Studying, studying, studying. I can’t give it my all. I’m sure I’m smarter than that, as I have always been, but now I feel like I am not the same. – Female from Syria, 21 years, arrival age 17.*

Academic achievement in the post-migration context is often linked to meeting basic existence needs and overcoming structural bottlenecks rather than to personal development or self-actualization. The participant also internalizes her functional impairment by questioning her intellectual ability. In doing so, she compares different versions of herself, highlighting a perceived decline from her previous level of functioning. Internal esteem is primarily satisfied at the level of growth needs. Thus, according to ERG, self-doubt or questioning one’s intellectual abilities is a sign of blocked growth needs.

##### Unmet relatedness need

In contrast to existence needs, the satisfaction of relatedness needs depends on others. The most frequently mentioned unmet needs in this subcategory included lack of communication skills and difficulties in forming friendships. Other unmet needs involved finding time for family and making good use of free time; these experiences are usually either driven by guilt or associated with feelings of guilt. One participant described how their anxiety inhibited their ability to meet their relatedness needs in the following way:

*It is difficult for me to manage everyday conversation with other students (I am a [name of university] student myself) without getting anxious. It is hard not to overthink, and I hate myself because I think I haven’t integrated well in the [name of country of residence] society – Female from Syria, 22 years, arrival age 13.*

The participant describes everyday peer interaction as a site where relatedness needs, such as belonging, acceptance, and reciprocal connection, are difficult to satisfy. What would typically be low-stakes social contacts (everyday conversation with fellow students) instead triggers anxiety, suggesting that interpersonal situations are appraised as evaluative. The participant’s emphasis on overthinking indicates rumination that likely operates both before and after interactions. She also articulates what these relational difficulties mean to her: they are not viewed as peripheral encounters but rather as indicators of how well she has integrated into society. Given that the participant comes from a collectivistic society, it is important for her to recognize herself as part of the social collective. Ultimately, this difficulty in satisfying relatedness needs is internalized as self-hate. The quote underscores the emotional weight that seemingly minor conversational encounters can carry for young refugees who are already vulnerable. It highlights how mental distress, in the form of anxiety, is embedded in a broader narrative of not belonging. The participant’s quote can also be interpreted through the lens of a blocked growth need. Her perceived failure to integrate into the host country’s society may have intensified her need to connect with her peers. The inability to satisfy the growth need for integration, combined with the failure to establish connections with peers, appears to have resulted in self-hate, reflecting what may be understood as a regressive motivational trap. As illustrated in the quote, the participant appears to have regressed from striving to fulfill her growth need of integrating into the host country and has instead become motivated to satisfy a relatedness need. However, she remains trapped in the frustration of being unable to satisfy either need.

### No unmet growth needs?

At face value, the youths’ responses suggest a lack of motivation to fulfill unmet growth needs. However, on closer examination, it may be argued that repeated blockage of growth need fulfillment has intensified their motivation to satisfy existence and relatedness needs. According to ERG theory, motivation does not diminish; rather, it is redirected toward other unmet needs. We propose the following application of the ERG model to the experiences of refugee youth, as illustrated in Figure 2. Refugees often experience a disrupted life trajectory as a result of war, displacement, and separation from loved ones. Such disruptions may constrain their ability to fulfill growth needs related to development, achievement, autonomy, self-confidence, and self-esteem. In turn, this may generate frustration-regression, thereby intensifying existence and relatedness needs. As needs are fulfilled or blocked, youth move along a continuum between motivational deficiency in relation to needs they have repeatedly failed to satisfy, and increased motivation to fulfill needs they have previously succeeded in satisfying.

## Discussion

The current study aimed to explore how young Arabic-speaking refugees conceptualize their mental health problems. Furthermore, it examined whether, and in what ways, these conceptualized problems affect daily functioning. Inductively, the mental health problems were organized into three categories: challenges within the self, difficulties in exile, and challenges in encounters with ‘the other’. Deductively, the impact on functioning was interpreted as unmet needs, categorized into existence needs and relatedness needs. No needs were identified as growth needs.

Two thirds of the responses were categorized as challenges within the self, suggesting that participants locate the responsibility for managing distress within themselves. This is the case even when they acknowledge external triggers, such as war, news, and legal status. One important aspect to consider is that young refugees may face structural barriers, such as having to work or study in order to obtain or maintain a residency permit in the host country. Thus, prioritization of mental health, defined as taking sick leave in order to seek professional help or reducing the working hours in order to accommodate psychological recovery, may have serious implications, such as deportation. In this sense, young refugees may be caught between taking care of their well-being and safeguarding their legal status. The most frequently mentioned problem within this category is concentration. Although attention difficulties are described in refugee mental health research, there are, to date, no attention-regulation interventions targeting refugees. This is important because concentration difficulties may reflect an expression of post-traumatic stress in the target group and can undermine daily functioning. Evidence suggests that intrusive memories involving involuntary, imagery-based impressions related to traumatic events (Ehlers et al., 2002) are associated with an inability to shift attention, attentional hijacking, and disruptions in concentration (Clark and Mackey, 2015; Holmes et al., 2017). Traumatic experiences prior to migration or during the journey contribute to psychological distress and functional disruption. However, the association between pre- and peri-migration traumatic experience, and trauma-related consequences, such as intrusions and concentration difficulties, maybe mediated by post-migration stressors (Cange et al., 2019; El-Awad et al., 2025). This underscores the deleterious impact of post-settlement stressors on refugees’ concentration (Renner et al., 2020). Furthermore, post-migration adversity may lead individuals to question whether the journey was worth the cost, and when this question is answered negatively, feelings of emotional suffering and mental distress may be intensified. Our findings extend this literature by underscoring how participants themselves place concentration difficulties at the center of their distress stories. Frustration with these difficulties comes as no surprise, given the high value placed on academic achievement as a survival path in the host country. The ability to concentrate and cope with studies becomes more than just a means of attaining personal development and prosperity; it becomes a way to survive and secure a future.

Interestingly, while trauma-related symptoms were mentioned by the participants, PTSD was rarely explicitly referred to. This is noteworthy given that epidemiological studies continue to report high prevalence rates of PTSD among refugees (Blackmore et al., 2020; Kien et al., 2019; Patanè et al., 2022; Tinghög et al., 2017). Even so, our findings echo those of previous qualitative studies (Aarethun et al., 2021; Renner et al., 2020). Although participants recognized symptoms consistent with PTSD, they did not label them as such. Younger refugees are more likely to use diagnostic terms for common mental illnesses compared to older refugees, suggesting an intergenerational difference in how mental health is conceptualized (Aarethun et al., 2021).

With regard to challenges in exile, involuntary loneliness was identified as the most common problem. Loneliness is increasingly recognized as a global health crisis, yet it is often overlooked in epidemiological studies of refugees (World Health Organization, 2025). Consistent with our findings previous research report the prevalence of loneliness in refugees between 15.9 and 47.7% (Nguyen et al., 2024). Several risk factors are noted to contribute to loneliness such as language barriers, separation from family and friends and feeling perceived as a ‘second class citizen’ (Nguyen et al., 2024). Furthermore, loneliness together with other post-migration factors have been reported as a stronger predictor of mental illness in refugees compared to pre-migration stressors such as traumatic events (Schweitzer et al., 2006). Our findings contribute to a deeper understanding of loneliness as a significant and common problem among refugees.

The encounter with ‘the other’ comprised post-migration stressors embedded in interpersonal relationships, such as family conflicts. With regard to family conflicts, one hypothesis suggests that children of immigrants undergo a process of “identity negotiation” during their identity-formation years (Ajrouch, 2004). This negotiation occurs as young people attempt to situate their sense of belonging along a spectrum, with the host country’s culture on one end and their parents’ culture on the other. Furthermore, a shift in power between parents and children after migration has been described as a parenting challenge that can create emotional distance within the family (Västhagen et al., 2022). Accordingly, studies suggest that families need resettlement services to support them in navigating changes in family structure and intergenerational relationships (Ayika et al., 2018). Therefore, psychosocial support for young refugees may benefit from including components that address interpersonal issues arising from generational gaps and help them navigate family dynamics through constructive intergenerational dialogues.

With regard to differences between Generation 1.5 and first-generation refugee youth, no differences were found at the category level. However, a closer examination of the subcategories suggests that Generation 1.5 more often conceptualizes difficulties related to staying on track than first-generation refugee youth. This may be due to higher mental health literacy, particularly regarding neuropsychiatric disorders such as attention deficit hyperactivity disorder. However, this hypothesis remains to be tested. In addition, Generation 1.5 appeared more likely to conceptualize mental health problems in terms of interpersonal conflicts and inner apprehensions. At the same time, the two groups were similar in several respects, particularly in their accounts of diminished vitality and emotional turbulence. Perhaps most notably, both groups seemed to experience isolation in relation to integration and socioeconomic demands to a similar extent. This similarity may reflect the shared effects of racism and discrimination, as subtle racialized comments and exclusionary experiences are unlikely to differ substantially between the two groups (Hirsch, 2019).

In the current study, we observed that participants expressed themselves using both culturally specific idioms and common Western expressions of mental distress, such as depression, anxiety, and panic attacks. This may indicate that the biopsychosocial model of mental distress, and its accompanying terminology, has, to some extent, been adopted by young refugee adults. However, it is essential to underscore that the biopsychosocial model does not, by any means, provide a comprehensive picture of how certain subgroups explain, express, and cope with their suffering. Earlier literature introduced the term “hyperdiversity” to describe multicultural urban environments in which diverse cultural groups coexist (Hannah, 2011). We extend this definition to include cognitive hyperdiversity, defined as the internal capacity to draw simultaneously on multiple terms and explanatory models of mental distress. Cultural cognitions from one’s culture of origin and from the host-country culture do not merely coexist; rather, they are intertwined and manifest in a dialectical manner. Thus, the activation of one does not deactivate the other. We argue that this cognitive hyperdiversity captures the experiences of Generation 1.5. Ultimately, young refugees’ conceptualizations of mental distress can be both culturally embedded and clinically legible at the same time. To meet the clinical needs of Generation 1.5, cultural sensitivity and openness must be prioritized.

Prior qualitative work suggests that causal attributions of distress may extend beyond biomedical or psychosocial explanations to include modernity-related and supernatural dimensions (Leavey et al., 2016). While the biopsychosocial model is useful for situating distress, the ecological model proposed by Miller and Rasmussen (2017) foregrounds displacement-related stressors such as unemployment, discrimination, family conflict, poverty, separation from family members, uncertainty regarding asylum status, and loss of social support. However, factors beyond the refugee journey and post-migration stressors, such as religiosity and spirituality, are treated only peripherally. Meaning-making resources that are salient in Arabic contexts, including spirituality and religiosity, may remain less visible in such accounts unless explicitly elicited. For example, maladaptive representations of religious beliefs have been associated with higher depressive and anxiety symptoms among university students in the United Arab Emirates (Shaban, 2024). In our data, a small number of participants referred to inner dilemmas involving religion and to existential ruminations, yet explicit references to spirituality or religiosity were otherwise scarce. One contextual explanation is that participants were enrolling in a setting dominated by psychotherapy, which may have encouraged psychological framings of distress in brief written responses. A second explanation may be generational: for some individuals in Generation 1.5, religiosity may be more embedded in family expectations and intergenerational dynamics than articulated as a primary explanatory model for personal distress. Importantly, the limited explicit mention of spirituality does not imply irrelevance; faith-based practices may still function as important coping resources. These considerations underscore the value of directly assessing spiritual concerns and coping when planning culturally responsive psychological interventions.

Similarly, only a small number of participants reported bodily strains. Although this was not systematically examined in the present analysis, intake interviews from the clinical trial indicated that some participants had previously sought care for somatic symptoms and were later informed that these complaints were linked to mental distress. This prior understanding may partly explain the relatively low proportion of explicitly reported bodily strains, compared to what might have been expected. Moreover, because participants were enrolling in a psychological intervention, they may have foregrounded difficulties they perceived as psychological rather than bodily. Lastly, the low proportion of reported bodily strains may further underscore the cognitive hyperdiversity characteristic of Generation 1.5.

In general terms, participants were more likely to bring up transdiagnostic symptoms. This is in line with previous research where Arabic expressions such as “*Ana taaban” (I’m tired), “Ana Khayfan” (I’m afraid”)* or *“Khouf” (fear)* and “Mou aader rakkezz” (I cannot concentrate) were used to communicate mental distress (Hassan et al., 2015). This suggests that symptom-specific interventions can be more beneficial than disorder/syndrome-focused interventions (Holmes et al., 2017). Cognitive behavioral therapy has proven to be effective in targeting individual transdiagnostic symptoms such as loneliness (Käll et al., 2021), low self-esteem (Berg et al., 2022), fear of giving birth (Rondung et al., 2018), procrastination (Rozental et al., 2018), and intrusive imagery memories related to trauma (Holmes et al., 2017). Other approaches such as the unified protocol is a transdiagnostic treatment which has proven to be effective in targeting various problems, including emotion dysregulation (Peláez et al., 2024) and neuroticism (Arunya et al., 2023). Acceptance and Commitment therapy (ACT) was for loneliness was evaluated among Persian divorced women (Mahmoudpour et al., 2021). Interpersonal psychotherapy (IPT) has shown to be effective for improving social functioning among individuals with depressive symptoms (Mahmoudpour et al., 2021). Low intensity interventions, such as the Friendship Bench could be suitable for refugee youth and young adults (Patena et al., 2025). Approaches such as Internal Family Systems Therapy can also be beneficial for refugee youth and young adults in terms of improving self-compassion and self-forgiveness (Buys, 2025), given that feelings of shame are common among the target population. Finally, psychodynamic therapy for fostering trust was evaluated among refugees with PTSD (Zinfandel and Svensson, 2024).

The deductive analysis based on Alderfer’s ERG theory indicated that participants’ unmet needs clustered into existence and relatedness needs, while no unmet growth needs were articulated. Regarding existence needs, difficulty meeting study-related demands was the most frequently reported, reflecting how education is experienced as a condition for stability and future security in the post-migration context. Regarding relatedness needs, participants commonly described lacking communication skills and struggling to build friendships, highlighting how everyday social interaction can be experienced as effortful and evaluative. These findings align with literature emphasizing that refugees’ mental health concerns are intertwined with broader social needs such as schooling, language, and integration resources (Arnold et al., 2015).

The absence of growth-oriented needs warrants further consideration. We propose that growth-oriented needs are not absent; rather, they are blocked by the disrupted life trajectories experienced by refugee youths. Academic achievement is usually treated as a growth need. However, our data suggest that it functions as an existence need, since several participants referred to studying in terms of learning the language of the host country, obtaining recognition of a foreign degree, or maintaining their residence status. For these reasons, we argue that academic achievement should be treated primarily as an existence need. This is not to say that academic achievement is solely an existence need. Needs are elastic, multilayered and intertwined. Another explanation is methodological. PSYCHLOPS elicits brief, problem-focused written responses and does not allow probing; thus, growth needs may be present but under-elicited in this format. A third explanation is contextual. The data may reflect a survivor mode, in which legal uncertainty, time-pressured academic demands linked to residency, and social isolation concentrate attention on stabilizing daily life and securing belonging, leaving limited opportunity to articulate growth-related needs. These explanations are not mutually exclusive. In either case, the finding is informative because it indicates what participants spontaneously prioritize when asked to identify problems and functional impact. Future research using in-depth interviews could examine whether growth needs emerge more clearly once existence and relatedness concerns become less acute. Taken together, ERG offers a useful lens for capturing the coexistence of existence and relatedness needs in forced migration contexts, where multiple need domains may be salient simultaneously (Alderfer, 1969).

### Limitations

The sample consisted primarily of highly educated individuals who self-referred to a clinical trial, which likely influenced both the types of problems expressed and the idioms used to describe distress. Therefore, the findings should be interpreted in light of their transferability to similar help-seeking, relatively educated Arabic-speaking refugee youth in comparable contexts. Previous research found no difference in mental health status between first and second generation Arab Australians (Kayrouz et al., 2015). However, first generation Arab Australians were more open to participating in digital mental health interventions compared to second generation Arab Australians (Kayrouz et al., 2015). Clinical terms, such as depression and anxiety, are likely familiar to this group, which may have shaped the way participants articulated their experiences. The use of PSYCHLOPS also introduced limitations, as its format generates brief written free-text responses. Unlike interviews, such responses cannot be probed for depth or clarification of meaning. Additionally, the context in which the data were collected represents a further limitation and may partly account for the absence of growth needs in the material. However, this should not diminish the value of an idiographic instrument for capturing participants’ own voices and priorities.

### Strengths

It comes as no surprise that PTSD assessment and intervention have dominated research on refugee mental health. While this has been valuable, it has also contributed to overshadowing of young refugees’ own perspectives on their mental health needs. This study contributes to an understanding of what young Arabic-speaking refugees identify as their primary mental health problems. Issues that have previously been neglected in psychotherapy research with refugees, such as concentration difficulties, loneliness, and family conflicts, should be taken into consideration when developing interventions for this population. To our knowledge, this is the first qualitative study to use an idiographic measure to explore the mental health problems of Arabic-speaking young refugees in Europe. The study taps into an underexplored area, which is that of the daily functioning of young refugees. Additionally, the study provides insight into the types of problems for which young refugees seek help. Nearly 60 % of the sample had lived in exile for 8–10 years, suggesting that a large proportion arrived as children. Generation 1.5 refers to individuals who arrive in the host country during childhood or adolescence and undergo their identity-formation years in exile (Benesch, 2008). This subgroup is often overlooked in the literature and thus a key strength of the present study is that it captures the voices of Generation 1.5. The research team members benefited from getting to know the participants beyond their PSYCHLOPS responses through the broader clinical trial. Follow-up calls allowed participants to elaborate on their distress, providing richer contextual understanding of the free-text data. The research team’s linguistic and cultural competence facilitated nuanced interpretation of culturally embedded idioms and expressions. Together, the cultural competence of the research team and the contextual framing of the study strengthened the fidelity of the phenomena explored in this research (Levitt et al., 2017).

### Clinical implications

The distress experienced by Arabic-speaking refugees extend beyond psychopathology and medicalization of human experiences. Clinically, this suggests that symptom-specific interventions may be particularly suitable for refugees. Such interventions include those targeting rumination, attention regulation, loneliness and communication skills. Social needs, such as academic support and assistance in forming new friendships, are relatively easy to address and can meaningfully improve the lives of young refugees in host countries. Such interventions would benefit from cultural adaptation in both language and components; for example, by using case examples drawn from real-life post-migratory experiences. Interventions focusing narrowly on post-traumatic stress may overlook what participants themselves identify as a primary source of distress. Lastly, family-sensitive components are generally recommended for the target group. Ultimately, upscaling the competencies related to cultural sensitivity is essential for meeting the needs of Generation 1.5.

## Conclusion

The study shows that young Arabic-speaking refugees most commonly describe mental distress through concentration difficulties, persistent fear and worry tied to legal and political uncertainty, loneliness, and relational tensions, rather than through diagnostic labels. This approach adds value by capturing what participants themselves prioritize; namely, concerns that may be underrepresented or missed in disorder-focused assessments, which underscores the need for culturally sensitive practices. Deductively, functional impacts translated into existence and relatedness needs. Growth needs did not emerge, suggesting either under-elicitation due to the nature of the utilized instrument or resettlement-focused functioning within the target group. The involvement of refugees in the development and implementation of psychosocial support and psychological interventions is crucial to ensure that their needs are genuinely addressed.

## Data Availability

The dataset contains confidential and sensitive information and is not publicly available due to privacy and ethical restrictions. Requests to access these datasets should be directed to gerhard.andersson@liu.se.
