# Supplementary material for: ‘A labyrinth with no way out’—the conceptualization of mental distress among Arabic-speaking refugee youth: a qualitative study
Source: Front Psychol. 2026 Jun 24;17:1735835. doi: 10.3389/fpsyg.2026.1735835 (PMC13345592; doi:10.3389/fpsyg.2026.1735835)
Supplement: Supplementary file 1 [file Table_1.DOCX]

| Appendix 2. Detailed Coding Scheme on the Conceptualization of Psychological Problems among Arabic-Speaking Refugee Youth and Young Adults | | | | | | | | |
| --- | --- | --- | --- | --- | --- | --- | --- | --- |
| **Categories** | **Challenges within the self** | **365** | **Difficulties in exile** | **80** | **Challenges in the encounter with ’the other’** | **32** | **Unmet needs** | **98** |
| **Subcategory** | **The inner apprehensions** | **107** | **Isolation in integration** | **57** | **Conflict with others** | **17** | **Unmet existence needs** | **70** |
| ***Codes*** | Social anxiety | 7 | Language barrier | 2 | Difficulties in taking criticism from others | 1 | Sleeping difficulties | 4 |
|  | Worry | 14 | Social alienation | 3 | Conflicts with family members | 3 | Poor hygiene | 3 |
|  | Fear | 21 | Difficulties in making new friends | 3 | Domestic psychological abuse | 1 | Poor appetite | 2 |
|  | Rumination | 18 | Acculturation stress | 4 | Challenges in regulating relationships | 1 | Coping with studies | 36 |
|  | Stress | 7 | Chosen isolation | 5 | Financial co-dependency | 1 | Coping with daily tasks | 14 |
|  | Anxiety | 15 | Negative perception regarding cultural differences | 2 | Conflict with neighbor | 1 | Coping with work-related tasks | 11 |
|  | Previous traumatic experiences | 7 | Separation from loved ones | 6 | Negative perceptions of the others | 9 |  |  |
|  | Panic attacks | 4 | Challenges with the formal migration status | 4 |  |  |  |  |
|  | Political stress | 8 | Involuntary loneliness | 22 |  |  |  |  |
|  | Existential rumination | 3 | Perceived racism | 1 |  |  |  |  |
|  | Inner delimma | 3 | Perceived injustice | 1 |  |  |  |  |
| **Subcategory** | **The diminished vitality** | **49** | **Socioeconomic demands** | **23** | **The longing for connection and stability** | **15** | **Unmet relatedness needs** | **28** |
| ***Codes*** | Loss of passion | 2 | Unemployment | 4 | Separation from romantic partner | 2 | Lack of communication skills | 14 |
|  | Depression | 12 | Difficulties in developing a daily routine | 7 | Negative couple conflict | 4 | Lack of time for the family | 2 |
|  | Loss of joy | 4 | Economic crisis | 2 | exploitation in the relationship | 1 | Failure in relationships | 1 |
|  | Indifference | 6 | Studies related difficulties | 8 | Bachelorship | 3 | Make good use of free time | 3 |
|  | Withdrawal | 4 | Challenges in the workplace | 2 | Imbalance in the responsibilities | 2 | Lack of the ability of developing friendships | 7 |
|  | Negative thoughts | 2 |  |  | Childlessness | 1 | Involuntary isolation | 1 |
|  | Despair | 9 |  |  | Low sexual desire | 1 |  |  |
|  | Meaninglessness | 5 |  |  |  |  |  |  |
|  | Helplessness | 1 |  |  |  |  |  |  |
|  | Hopelessness | 1 |  |  |  |  |  |  |
| **Subcategory** | **The emotional turbulence** | **29** |  |  |  |  |  |  |
| ***Codes*** | Anger | 7 |  |  |  |  |  |  |
|  | Shame | 1 |  |  |  |  |  |  |
|  | Emptiness | 4 |  |  |  |  |  |  |
|  | Irritability | 4 |  |  |  |  |  |  |
|  | Grief | 3 |  |  |  |  |  |  |
|  | Envy | 1 |  |  |  |  |  |  |
|  | Guilt | 1 |  |  |  |  |  |  |
|  | Dissociation | 4 |  |  |  |  |  |  |
|  | Feeling of insecurity | 2 |  |  |  |  |  |  |
|  | Emotional lability | 2 |  |  |  |  |  |  |
| ***Subcategory*** | **The eroded self** | **39** |  |  |  |  |  |  |
|  | Self-hate | 1 |  |  |  |  |  |  |
|  | Low self-esteem | 5 |  |  |  |  |  |  |
|  | Perceived worthlessness | 3 |  |  |  |  |  |  |
|  | Low self-confidence | 12 |  |  |  |  |  |  |
|  | Negative view of self-realization | 9 |  |  |  |  |  |  |
|  | Self-criticism | 2 |  |  |  |  |  |  |
|  | Self-alienation | 1 |  |  |  |  |  |  |
|  | Self-dissatisfaction | 5 |  |  |  |  |  |  |
|  | Perceived lack of freedom | 1 |  |  |  |  |  |  |
|  | Perceived carelessness | 1 |  |  |  |  |  |  |
| **Subcategory** | **Difficulties staying on track** | **100** |  |  |  |  |  |  |
| ***Codes*** | Mental rigidity | 1 |  |  |  |  |  |  |
|  | maladaptive daydreaming | 2 |  |  |  |  |  |  |
|  | Impulsivity which causes troubles | 3 |  |  |  |  |  |  |
|  | Lack of motivation | 4 |  |  |  |  |  |  |
|  | Concentration difficulties | 52 |  |  |  |  |  |  |
|  | Forgetfulness | 3 |  |  |  |  |  |  |
|  | Perceived learning difficulties | 5 |  |  |  |  |  |  |
|  | Procrastination | 2 |  |  |  |  |  |  |
|  | Shortcomings in planning and execution abilities | 5 |  |  |  |  |  |  |
|  | Lack of spatial perception | 3 |  |  |  |  |  |  |
|  | Inner restlessness | 3 |  |  |  |  |  |  |
|  | Absent-minded | 3 |  |  |  |  |  |  |
|  | Perceptual sensitivity | 2 |  |  |  |  |  |  |
|  | Unhelpful outer behaviors | 7 |  |  |  |  |  |  |
| **Subcategory** | **The bodily strains** | **41** |  |  |  |  |  |  |
| ***Codes*** | Insomnia | 8 |  |  |  |  |  |  |
|  | Tiredness | 6 |  |  |  |  |  |  |
|  | Loss of energy | 12 |  |  |  |  |  |  |
|  | Binge eating | 1 |  |  |  |  |  |  |
|  | Bad appetite | 3 |  |  |  |  |  |  |
|  | Swallowing problems | 1 |  |  |  |  |  |  |
|  | Irritable bowel syndrome | 1 |  |  |  |  |  |  |
|  | Autonomic symptoms of anxiety | 9 |  |  |  |  |  |  |
